# Protocol for the Controlled evaLuation of Angiotensin Receptor blockers for COVID-19 respIraTorY disease (CLARITY): a randomised controlled trial

**DOI:** 10.1186/s13063-021-05521-0

**Published:** 2021-08-28

**Authors:** Carinna Hockham, Sradha Kotwal, Arlen Wilcox, Abhinav Bassi, James McGree, Carol Pollock, Louise M. Burrell, Nikita Bathla, Mallikarjuna Kunigari, Vinay Rathore, Michael John, Enmoore Lin, Christine Jenkins, Angus Ritchie, Andrew McLachlan, Thomas Snelling, Mark Jones, Vivekanand Jha, Meg Jardine, on behalf of the CLARITY Investigators

**Affiliations:** 1 The George Institute for Global Health, University of New South Wales, Sydney, Australia; 2 The George Institute for Global Health, Imperial College London, London, UK; 3 Prince of Wales Hospital, Sydney, Australia; 4 NHMRC Clinical Trials Centre, University of Sydney, Sydney, Australia; 5 The George Institute for Global Health, New Delhi, India; 6 Queensland University of Technology, Brisbane, Australia; 7 Royal North Shore Hospital, Sydney, Australia; 8 Kolling Institute of Medical Research, University of Sydney, Sydney, Australia; 9 Department of Medicine, The University of Melbourne, Austin Health, Heidelburg, Victoria Australia; 10 All India Institute of Medical Sciences, Raipur, India; 11 Christian Medical College, Ludhiana, India; 12 Concord Repatriation General Hospital, Sydney, Australia; 13 The University of Sydney, Sydney, Australia; 14 Sydney School of Public Health, University of Sydney, Sydney, Australia; 15 The Sydney Children’s Hospitals Network, Westmead, NSW Australia

**Keywords:** COVID-19, Angiotensin receptor blockers, Renin-angiotensin system, RCT, Bayesian adaptive design

## Abstract

**Background:**

SARS-CoV-2 binds to membrane-bound angiotensin-converting enzyme 2 (ACE2) which may result in downregulation of membrane-bound ACE2. ACE2 is a key regulator of the renin-angiotensin system (RAS) and is responsible for degrading angiotensin II and thereby counteracting its pro-inflammatory, pro-fibrotic effects mediated through the angiotensin II type 1 receptor (AT1R). As AT1R is directly blocked by angiotensin receptor blockers (ARBs), these agents may offer a safe, low-cost solution for reducing COVID-19 respiratory outcomes.

**Methods and discussion:**

CLARITY is a pragmatic, adaptive, two-arm, multi-centre, comparative effectiveness phase III randomised controlled trial that examines whether ARBs reduce COVID-19 severity among high-risk patients. Recruiting in India and Australia, the trial will compare treatment with a maximum tolerated daily dose of an ARB to standard of care. Treatment allocation is blinded in India but open-label in Australia due to interruptions to placebo supply in the latter. The primary endpoint is a 7-point ordinal scale of clinical states, ranging from no limitation of activities (category 1) to death (category 7), assessed on day 14. Secondary outcomes include the 7-point scale assessed at day 28 and 28- and 90-day mortality. The design adapts the sample size based on accumulating data via frequent interim analyses and the use of predictive probability to determine whether the current sample size is sufficient or continuing accrual would be futile. The trial commenced recruitment on 18 August 2020.

**Trial registration:**

ClinicalTrials.gov, NCT04394117. Registered on 19 May 2020. Clinical Trial Registry of India: CTRI/2020/07/026831)

**Supplementary Information:**

The online version contains supplementary material available at 10.1186/s13063-021-05521-0.

## Article summary

### Strengths and limitations of this study

- CLARITY will test the effectiveness of repurposing a widely available class of medications for the treatment of COVID-19.
- The risk of COVID-19 transmission is minimised by restricting trial-specific in-person encounters between participants and health care workers.
- The trial burden on sites is reduced by aligning trial processes with routine clinical care and targeting study data collection to measures of COVID-19-related effectiveness and selected safety endpoints.
- Adaptive Bayesian sample size re-estimation methodology are employed to protect the trial from an indeterminate result, an approach chosen because of the absence of information on potential effect size.
- The unavoidable use of open-label study medication in Australia is a limitation but is offset by the inclusion of a large number of sites in India (where placebo is available) and mitigated by the blinding of the central study team, including trial statisticians.

## Background

Coronavirus disease 2019 (COVID-19) is a recent and one of the deadliest examples of cross-species viral transmission affecting the human population. By the end of July 2021, COVID-19 has resulted in more than 190 million confirmed cases and 4.1 million confirmed deaths worldwide [1, 2]. Its clinical spectrum is broad, ranging from asymptomatic infection to respiratory failure, multi-organ dysfunction, and death [3–7]. Risk factors for very severe disease, which generally manifests as acute respiratory distress syndrome (ARDS), interstitial pneumonia and/or sepsis, consistently include male sex; older age (≥ 60 years) [8, 9]; pre-existing comorbidities, such as hypertension, diabetes, heart failure, chronic kidney disease, and chronic respiratory illness [10]; and obesity (body mass index [BMI] ≥ 30 kg/m^2^) [11, 12].

Whilst there have been unprecedented advances in public health and clinical strategies, therapeutics and vaccine development for reducing the impact of COVID-19, outbreaks of the severe acute respiratory syndrome coronavirus 2 (SARS-CoV-2) pandemic continue, with emerging variants adding to uncertainty on its curtailment [13–15]. Widely available, low-cost effective treatments that can be easily and quickly implemented in diverse settings would help ameliorate outbreaks whilst more definitive solutions are found.

SARS-CoV-2 causes COVID-19 through binding to angiotensin-converting enzyme 2 (ACE2) [16, 17], a transmembrane protein and key regulator of the renin-angiotensin system (RAS) [18]. Cell entry through binding to ACE2 has been demonstrated for SARS-CoV-2 in in vitro studies [16, 18] and for the homologous SARS-CoV-1 [19] in a preclinical model [20]. Viral-binding of ACE2 may lead to downregulation of membrane ACE2, causing a dysregulated local RAS that favours inflammation and ongoing tissue damage secondary to excess angiotensin II [21, 22]. Systemic effects are also possible because the dysregulated local RAS is associated with prolonged shedding of the catalytically active site of ACE2 into the circulation [22]. The angiotensin II type 1 receptor (AT1R) may play a key role in COVID-19 pathophysiology as the normal physiological process of ACE2 receptor-mediated endocytosis is dependent on the AT1R and a number of the downstream steps involved in the dysregulation of the local RAS may be mediated by the AT1R [23]. Correlative evidence is provided by the finding that AT1R blockers (angiotensin receptor blockers [ARBs]) reverse the inflammatory impact of coronavirus spike-protein binding to ACE2 in a preclinical model of SARS-CoV-1 disease [20].

ARBs are a commonly prescribed, widely available and affordable class of medications that are used to treat a range of chronic conditions, including hypertension, heart failure, and chronic kidney disease [24]. Observational data suggest that treatment with ARBs could be protective in viral pneumonias, including influenza [25]. The majority of evidence on the role of RAS inhibitors (ARBs and ACE inhibitors) in COVID-19 has been derived from reports of outcomes in prevalent users of RAS inhibitors, both from observational studies and from randomised controlled trials [26–28]. Two reported open-label trials randomised prevalent users of RAS inhibitors contracting COVID-19 to continuation or cessation. The BRACE CORONA trial found that continuation or cessation of treatment with RAS inhibitors did not affect the number of days alive and out of hospital or 30-day mortality in 659 participants hospitalised with COVID-19 [29]. Similarly, the REPLACE COVID trial, which randomised prevalent users of RAS inhibitors with COVID-19 to either continue or discontinue RAS inhibitor use, reported no difference on a global rank score of COVID-19 severity or the need for intensive care admission or mechanical ventilation in 152 participants [30]. These trials suggest that continuing RAS inhibition is safe in participants hospitalised with COVID-19 disease who are already established users of RAS inhibitors. The trials are valuable but do not directly address the question of whether the introduction of RAS inhibition in RAS inhibition-naïve patients would improve outcomes in COVID-19. Differences in the two scenarios include the timing of RAS inhibition relative to SARS-CoV-2 exposure, the establishment of RAS inhibition effects during early disease, and the time course of the wash-out of RAS inhibition and the associated physiological effects.

By directly blocking the AT1R and reducing the tissue-damaging effects of angiotensin II, we hypothesise that ARBs will reduce the severity of COVID-19 respiratory clinical outcomes. The Controlled evaLuation of Angiotensin Receptor blockers for COVID-19 respIraTorY disease (CLARITY) trial will test whether treatment with an ARB in addition to standard of care improves clinical outcomes in high-risk COVID-19 patients, compared to standard care alone using a pragmatic trial design.

## Methods/design

### Design overview

CLARITY is a pragmatic, adaptive, two-arm, multi-centre, comparative effectiveness phase III randomised controlled trial, conducted in India and Australia, designed to evaluate whether ARBs reduce the severity of COVID-19 among high-risk patients. The trial, designed in accordance with SPIRIT guidelines, will recruit patients with COVID-19 deemed to be at high risk of severe illness (Supplementary Material—SPIRIT Checklist) [31]. Overarching principles informing study design include (i) minimising the trial burden on clinical staff and participants, for example, through the alignment of trial processes with routine clinical care and prioritisation of essential routinely collected data that can be extracted from existing medical records, (ii) minimising trial-mandated in-person encounters that could increase the risk of exposure and transmission, and (iii) improving research efficiency by incorporating adaptive sample size re-estimation methodology with pre-defined rules and processes, an approach chosen given the absence of informative existing data on the potential effect of the intervention.

### Study setting

The trial is designed to enrol participants at risk of severe disease and is being conducted across 25 hospital sites in India and Australia (Supplementary Material—List of CLARITY Investigators and study sites), in line with local standard clinical processes. As a result, in India, participants are recruited from sites providing inpatient care only. In Australia, participants are recruited from trial sites providing inpatient care as well as sites providing regular monitoring and management of community-based patients. The majority of study recruitment is now expected to take place in India given the relatively larger number of people affected with COVID-19 in India.

### Eligibility criteria

The eligibility criteria are broadly designed to recruit participants with active COVID-19 who are at risk of severe disease. Risk of severe disease is deemed to be hospital admission for COVID-19 management or the presence of least one risk factor for severe disease in specialised units managing patients in the community (Australia only) (Table 1). Exclusion criteria are designed to avoid enrolling patients who are at risk of not tolerating the medication, of experiencing adverse effects, or who would be ineligible for use of the ARBs for other indications.

**Table 1:** Eligibility criteria, according to hospital admission status at time of enrolment

Criteria	Inpatients (India and Australia)	Virtual care patients (Australia only)
*Inclusion*		
Laboratory-confirmed active SARS-CoV-2 infection within 10 days prior to randomisation, using any locally approved testing method	✓	✓
Aged ≥ 18 years	✓	✓
Be at high risk of severe disease, defined as:		
• Requiring hospital admission for the management of COVID-19 or,	✓	❌
• Having at least one of the following risk factors for severe disease: - Aged ≥ 60 years - BMI ≥ 30 kg/m^2^ - Diagnosis of diabetes defined as HbA1c ≥7% *and/or* use of glucose-lowering medication - History of cardiovascular disease^a^ - History of chronic respiratory disease^a^ - Current treatment with immunosuppression	±	✓
SBP ≥ 120 mmHg, *or* SBP ≥ 115 mmHg and receiving treatment with a non-RAAS-inhibitor blood pressure-lowering agent that can be ceased	✓	✓
Willing and able to perform trial procedures	✓	✓
*Exclusion*		
Reduced eGFR in preceding 3 months, defined as < 30 mL/min/1.73m^2^ or the absence of an eGFR test	✓	❌
Reduced eGFR in preceding three months, defined as < 45 mL/min/1.73m^2^ or the absence of an eGFR test	❌	✓
Elevated serum potassium, defined as > 5.2 mmol/L, within the preceding 3 months or the absence of a serum potassium test	✓	✓
Receiving treatment with an ACE inhibitor, ARB, aldosterone antagonist, aliskiren or angiotensin receptor neprilysin inhibitor	✓	✓
Known intolerance to ARBs	✓	✓
Known symptomatic postural hypotension	✓	✓
Known biliary obstruction or severe hepatic impairment	✓	✓
Inability to take medications by mouth during the first 48 h after randomisation	✓	✓
Women who are currently pregnant or breast feeding (India) *Or* Women < 51 years without a negative pregnancy test during the previous 3 days and/or who do not agree to use adequate contraception during the 28-day treatment period (Australia)	✓	✓

^a^As defined by the treating clinician

Patients with an estimated Glomerular Filtration Rate (eGFR) < 30 mL/min/1.73m^2^ or a serum potassium > 5.2 mmol/L within 3 months prior to randomisation, or an absence of the relevant tests, are excluded. It is expected that most patients requiring hospital care or with the defined high-risk conditions will have had serum creatinine and potassium tests conducted upon admission as part of routine care or in the 3 months prior to diagnosis. Patients without such tests are ineligible to participate. In keeping with the principle of designing the trial so that its requirements align with those of routine care, a higher eGFR threshold for community-based patients is used to avoid a requirement for additional clinical monitoring and face-to-face interactions in community-based patients.

### Informed consent, recruitment and randomisation

Prospective patients are approached by the site’s principal investigator or study co-ordinator and provided with a written information sheet, physically or electronically, that remains with them for infection control reasons. Informed consent is obtained verbally either via audio-recording or with appropriate documentation by an independent, third-party witness.

Randomisation takes place within 10 days of the confirmed SARS-CoV-2 diagnosis, using a central interactive web response system that contains a computer-generated randomisation schedule. Participants are randomly assigned in a 1:1 ratio to standard of care plus an ARB or standard of care with or without matched placebo (India and Australia, respectively), stratified by country. In Australia, further stratification is performed by baseline hospital admission status (i.e. admitted to hospital or managed virtually in the community). Unless they withdraw their consent, participants who prematurely discontinue study treatment will continue to be followed according to intention-to-treat (ITT) principles.

Participant recruitment is reviewed weekly by the research team. A close ongoing relationship has been maintained with all sites including regular meetings, newsletters, and phone calls. Challenges associated with recruitment are discussed and troubleshooted at each Executive Trial Steering Committee meeting held monthly.

### Trial intervention, blinding, and dose management

The intervention is a daily dose of an ARB for 28 days. The intention at the time of study design was for placebo control where possible, although open label was deemed an acceptable alternative and preferable to not conducting the study at all. In India, where placebo could be sourced, the study intervention is telmisartan, supplied as 40 mg tablets or matching placebo. In Australia, placebo was not available at the time of study commencement due to COVID-19-related interruptions to supply chains and the study was initiated open-label with an intention to pursue placebo procurement if it became available. Australian Principal Investigators were given information on the half-life, receptor binding affinities and pressor effect for common ARBs, and were permitted to select an ARB as per the hospital formulary (detailed in the study Guidance document).

The central study team remain blinded to study allocation in both countries throughout the course of the trial. In India, both the participant and the treating site staff are also blinded to treatment allocation. A separate, unblinded statistician will conduct the pre-planned interim analyses required for reporting to the independent Data Safety Monitoring Board (DSMB).

In both countries, treating practitioners are encouraged to commence treatment with a low to moderate starting dose (or placebo) and to increase if tolerated. Study sites are provided guidance on ARB prescribing, monitoring, and adjustment derived from national educational material in the public domain [32, 33]. Individual management decisions on dose initiation, titration, symptom and safety monitoring are at the discretion of the treating team. In the event that prescribing practices are inconsistent with standard guidance, a query will be raised by the study team, but will not constitute a protocol deviation. The study is designed as an effectiveness rather than efficacy trial on the rationale that the widespread use of ARBs and the experience of practising clinicians in the study centres with their management means a true effectiveness approach can be tested.

Adherence to ARB prescription and records on daily dosing will be verified using medical records by the study staff. Treating clinicians are encouraged to manage blood pressure according to their usual practices with the caveat that they avoid prescription of ARB and they prioritise cessation or down-titration of concomitant medications rather than study drug for the management of low blood pressure. Daily monitoring of blood pressure of all study participants is recommended. In Australia, community-based participants who are being managed virtually at the time of randomisation are provided an electronic blood pressure monitor and instructed on its use. Clinicians and patients are advised to avoid non-steroidal anti-inflammatory agents (NSAIDs) during the 28-day treatment period. Regular monitoring of serum potassium is recommended for inpatients, as would be consistent with routine clinical care for hospitalised patients.

### Unblinding procedures (India only)

In the event that knowledge of the treatment will significantly influence a participant’s clinical management, emergency unblinding will be permitted via a 24-h emergency unblinding phone line staffed by an authorised representative. The decision to unblind ultimately rests with the Principal Investigator at the site or the treating/attending clinician.

### Primary outcome

The primary outcome is a 7-point ordinal categorical scale of clinical outcomes, assessed on day 14 (Table 2), modelled via a proportional odds cumulative logistic regression model, and the treatment difference will be assessed via the log-odds. This is a modified version of the 9-point scale recommended by the WHO Research and Development Blueprint expert group and has been used in other COVID-19 trials [34]. The scale captures a broad spectrum of COVID-19 clinical severity and can be derived from the medical record or by phone calls with participants. The scale used in the CLARITY trial was simplified as the original scale includes a category for uninfected with no viral DNA as well as asymptomatic with viral RNA detected. These categories were omitted because viral RNA collection was not mandated in the trial to avoid additional patient encounters beyond routine care.

**Table 2:** Seven-point ordinal categorical primary endpoint of the CLARITY trial

Category	Description
1	Not hospitalised with no limitation on activities
2	Not hospitalised with some limitation on activities
3	Hospitalised, not requiring supplemental oxygen
4	Hospitalised, requiring supplemental oxygen
5	Hospitalised, requiring non-invasive mechanical ventilation or high-flow nasal cannular (HFNC) therapy
6	Hospitalised, requiring mechanical ventilation ± additional organ support
7	Died

### Secondary and exploratory outcomes

Secondary and exploratory outcomes are selected because they reflect the clinical severity of COVID-19 disease or are well-known adverse events related to ARBs. They include the 7-point ordinal scale assessed at day 28; 28- and 90-day all-cause mortality (binary outcome); any requirement for intensive care unit (ICU) admission between baseline and days 28 and 90 (binary outcomes); median length of ICU stay, in days, between baseline and day 90; any occurrence of respiratory failure (defined as requirement for non-invasive or invasive mechanical ventilation) (binary outcome); occurrence of kidney failure (defined as the requirement for dialysis) (binary outcome); median number of dialysis days between baseline and day 28; median length of hospital stay, in days, between baseline and days 28 and 90; median number of ventilator-free days between baseline and days 28; presence or absence of acute kidney injury (AKI); and presence or absence of hypotension requiring vasopressors.

Exploratory outcomes include any occurrence of hyperkalaemia between baseline and day 28 (defined as any serum potassium result > 6.0 mmol/L) (binary outcome) and the proportional outcome of oxygen (O_2_) saturation/fraction of inspired (FiO_2_) at days 8 and 14.

### Safety monitoring

Other than the pre-specified outcomes, serious adverse events will not be collected in recognition of the repurposing nature of the trial. The safety profile of ARBs has been well-established in large, randomised trials, and the size of this study will be insufficient to establish whether the safety profile in the COVID-19 setting is significantly different from that already observed in non-COVID-19 settings. The safety events of hypotension, hyperkalaemia, or increases in serum creatinine will be collected as secondary outcomes. These events were selected as well-described events associated with ARB use.

### Follow-up schedule

**Fig. 1:**
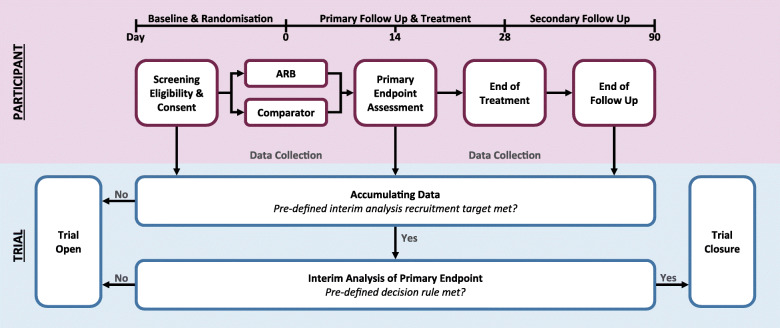
Trial overview and participant schedule

**Fig. 2:**
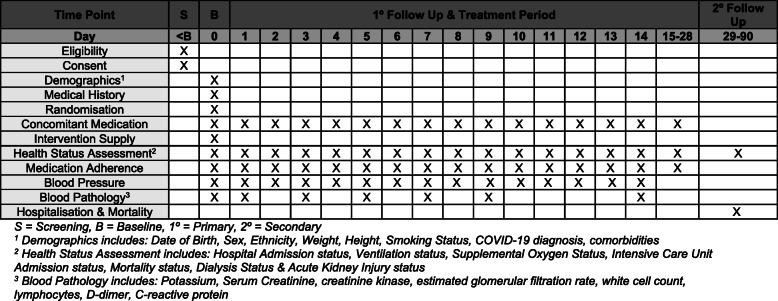
Participant assessments

Participants are followed up daily between day 0 and day 28, and once at day 90 (Figs. 1 and 2).

The trial does not mandate pre-specified face-to-face visits or additional biochemical testing specific to ARBs beyond that required as part of routine clinical care. This is in accordance with the effectiveness nature of the trial and the rationale that the well-known safety profile and familiarity of these medications does not warrant the infection risk related to visits that are not essential for clinical care. For the most part, participants will not be followed up outside of routine care, unless additional information on blood pressure, medication adherence, and clinical health status is required. This will be performed remotely, via telephone contact only.

### Data collection

De-identified trial data will be collected directly from patients’ medical records and entered electronically into trial-specific electronic case report forms (eCRFs). Where appropriate, these data will be supplemented with information provided by the treating staff or participants themselves (e.g. information relating to medication adherence and blood pressure). The eCRFs are housed within a REDCap (Research Electronic Data Capture) database [35, 36], a secure, web-based application, hosted on secure servers in India and Australia, respectively, and backed up daily. All trial data will be stored in keeping with local regulatory requirements and accessed only by approved study personnel.

Data that are essential for assessing the primary outcome and relevant secondary outcomes will be collected daily for the first 28 days. This includes information on blood pressure, dialysis requirement, any serum creatinine and serum potassium levels, medication adherence, and clinical health status on the 7-point ordinal scale. The timing of serum creatinine and serum potassium testing will be determined by treating staff and will be monitored by study staff. As above, safety monitoring practices that are inconsistent with standard guidance will be queried by the study team but will not constitute a protocol deviation. All other secondary and exploratory outcomes will be extracted from the patients’ medical records after days 28 and 90. In the event that a patient is discharged from hospital or stops receiving virtual care prior to day 14, follow-up of the primary outcome will be performed via telephone contact. Other secondary outcomes will be collected from patient’s medical records or via telephone contact after day 90. No biological specimens will be collected as part of the trial; however, the study consent includes an optional consent to retain, store, and use leftover blood samples obtained during routine clinical care for further research, for another, separate, ethically approved research project based at one Australian site (Austin Health). Adjudication of study endpoints will not be performed.

### Statistical considerations

In the absence of prior information on the potential effect size of ARBs on COVID-19 severity, the trial will follow adaptive sample size re-estimation principles. This approach protects the trial against an indeterminate result, whilst avoiding over-sampling. The study uses Bayesian methods to model the outcomes of interest at pre-planned interim analyses. All inferences will be based on the joint posterior distribution of the model parameters, and this distribution will be sampled using Markov chain Monte Carlo methods.

#### Primary and secondary analyses

The estimated treatment effect on the primary endpoint will be expressed as the common odds ratio, corresponding to the odds of a better outcome in the ARB versus the comparator arm on the 7-point ordinal scale at day 14 and its 95% credible interval. This will be modelled using a proportional odds logistic regression model [37], further details of which are provided in the statistical analysis plan, which will be published separately. The assessment of the primary endpoint at day 14 reflects the timeframe within which most SARS-CoV-2-positive patients have either developed severe disease or begun to recover [3].

The primary analysis will be an ITT analysis, whereby comparisons will be made between all participants randomised to the treatment arms irrespective of whether they received or completed their course of allocated treatment.

Secondary analyses will comprise assessment of the primary endpoint in patients who were randomised and treated as per the trial protocol (i.e. per-protocol population), as well as analyses of pre-specified secondary and exploratory outcomes, using statistical models that are appropriate for the specific outcome.

Analyses of the primary endpoint will be Bayesian. Details of these approaches, and how missing data will be handled, are described in the statistical analysis plans

#### Sample size, interim analyses, and stopping rules

Prior to the start of the trial, trial simulations were performed to evaluate the trial operating characteristics under a range of scenarios, data configurations, interim timings, and decision thresholds (1000 simulations for every scenario). A maximum sample size of 2200 was applied. The simulations showed that, across a variety of scenarios, the trial is expected to yield a power > 80% if the odds ratio for the intervention is ≥ 1.25, with the chance of declaring a false positive being < 0.08. Of note, the average sample size required to achieve this was considerably lower than the imposed maximum sample size of 2200. Further details of these simulations, the different scenarios considered, and their underlying assumptions will be provided in the statistical analysis plan prior to the first scheduled interim analysis.

The first interim analysis will be conducted 14 days after the 700th participant has been enrolled. Thereafter, an interim analysis will be conducted approximately 14 days after every 300 additional participants have been enrolled (to ensure follow-up is reached for the last enrolled patient) until the trial stops or the maximum sample size of 2200 has been reached. All interim analyses will be based on the primary outcome. At each interim analysis, the predictive probability that the intervention is effective, or that the trial is futile, will be estimated and assessed against pre-defined decision thresholds (Table 3). In both cases, the predictive probability of success will be computed by averaging over the predictive distribution of the response. For effectiveness, this will be calculated for participants who have been randomised but not yet responded, or who have completed their 14-day treatment period. If the predictive probability is greater than the decision threshold of 95% (Table 3), then a recommendation is put forward to the DSMB that the trial be stopped for effectiveness. For futility, the calculation is performed for the participants who have been randomised but not yet responded as well as those who have not been enrolled, up to the maximum sample size of 2200. If the predictive probability indicates that fewer than 2% of the trials detect a treatment benefit, a recommendation is put forward to the DSMB that the trial be stopped for futility.

**Table 3:** Pre-specified decision rules for the CLARITY trial. Details about how these probabilities are evaluated/approximated are available in the statistical analysis plan

Reason for stopping	Posterior probability	Decision rule
Effectiveness	Pr(OR < log(1.00)) > 0.975	> 0.95
Futility	Pr(OR < log(1.00)) > 0.975	< 0.02

### Study oversight

Oversight of the CLARITY trial is provided by the Trial Steering Committee. A Consumer and Community Engagement Committee assists the Trial Steering Committee, providing advice and feedback on trial design and participant-facing documents. An independent DSMB is responsible for reviewing interim analysis results in a unblinded manner and advising on overall participant safety.

The Trial Steering Committee Executive Committee provides additional supervision of the operational aspects of the trial. Within the pandemic setting, limitations are placed on hospital and health research systems limiting in-person interactions. On-site monitoring will not be performed for CLARITY, remote monitoring and auditing will be performed.

### Interactions with other trials

In the current pandemic setting, clinical sites may have multiple research projects being conducted that require patient participation. CLARITY encourages co-enrolment in other trials (provided that the intervention does not relate to RAS inhibition) in order to maximise the generation of evidence. Sites are encouraged to coordinate recruitment approaches for trials involving participants with COVID-19.

### Ethical considerations and dissemination

The CLARITY trial will be conducted in accordance with the principles of the Declaration of Helsinki, the National Ethical Guidelines for Biomedical and Health-Related Research Involving Human Participants in India and the National Statement on Ethical Conduct in Human Research in Australia. The study has received ethical approval from The George Institute for Global Health Ethics Committee in India (ref. no. 14/2020) and the Sydney Local Health District Ethics Review Committee (Royal Prince Alfred Hospital Zone) (Code: EC0113) in Australia (ref. no. X20-0118 & 2020/ETH00742). The trial design was informed by specific guidance including ethical principles relevant to the conduct of COVID-19 trials [38, 39]. Any substantial changes to the protocol will be submitted to the primary approving ethics committee in each country for approval and then communicated with sites and investigators in line with local regulatory processes.

A pre-formatted template has received pre-approval by the relevant Ethics committees to allow rapid dissemination of results to surviving participants at the conclusion of the trial (Supplementary Material—CLARITY Trial Results Template). The results of the trial will be submitted for publication in international peer-reviewed journals and for presentation at national and international conferences.

### Trial status

The protocol version is number 3.0, dated 29 October 2020. The trial commenced recruitment on 18 August 2020 and is ongoing.

## Discussion

The COVID-19 pandemic is now in its second year. The discovery of effective vaccines has been a very anticipated and welcome development. Some therapies have been found to improve outcomes including corticosteroids [40, 41], the IL-6 receptor antagonists, tocilizumab and sarilumab [42, 43], and the anti-viral agent, remdesivir [44]. However, these are not universally available and importantly have not eliminated the disease. With only a minority of the world’s population vaccinated, the emergence of multiple viral variants and the predictions of secondary and tertiary waves of infection unfortunately realised, more treatments are needed for COVID-19 disease. Investigating existing medications with a known safety profile and widespread availability in a scientifically robust manner offers an efficient approach to finding treatments that can be rapidly and widely implemented. The CLARITY trial is a pragmatic, minimally intrusive trial that aims to achieve this.

Observational data have shown that prevalent use of ARBs at the time of contracting COVID-19 is associated with either better or similar outcomes compared with people who are not receiving ARB treatment [26]. Moreover, there is randomised evidence that continuation versus withdrawal of RAS inhibitors in COVID-19 patients with an existing indication for these medications does not impact the clinical outcome of COVID-19 [29, 30]. Whilst the withdrawal of these agents does not appear to worsen the course of COVID-19 disease, the addition of RAS blockade may yet improve outcomes due to factors such as presence of blockade at the time of infection, pharmacokinetic wash-out time, and physiological time to response. There is currently no randomised evidence for the effect of the initiation of ARBs on the course of disease in participants with newly diagnosed COVID-19 disease [27]. CLARITY is one of 12 trials registered on ClinicalTrials.gov (as of March 18 2021) that will test this hypothesis. Individually or in aggregate, these trials promise to shed light on the ability of RAS blockade to improve outcomes in COVID-19 disease.

The experience of research in the pandemic has generated some lessons for the conduct of research in general. The pandemic has highlighted the extent to which well-designed and supported research and health systems can respond to an unanticipated need for evidence generation. The UK has stood out for its success in generating evidence for the clinical management of COVID-19 disease. The established research and health infrastructure of the National Institute for Health Research (NIHR) embedded within the National Health Service (NHS) [45] has meant trials could be, and were, rapidly deployed using pre-existing systems. As a result, the NIHR has supported a total of 91 COVID-19 studies [46], including the well-known RECOVERY trial, which has been at the forefront of COVID-19 evidence generation. The clear illustration of the clinical benefits that can be derived from research systems embedded in clinical practice will hopefully inspire health system custodians long after the pandemic has passed.

The pandemic setting has also inspired review by oversight bodies on the necessity and justification of common trial features [47–49]. The result has been an increase in the uptake of recent initiatives such as virtual study visits and streamlined trial procedures [50, 51]. Review of the success of these measures will hopefully inspire the greater adoption of technology to reduce the burden of research participation on patients and lead to greater research efficiency.

Lastly, there has been the development of specific guidance on the ethical principles informing the design and conduct of COVID-19 research, such as requirements to consider the impact of COVID-19 research on participant safety, on potentially strained health systems and on the safety of research staff [39]. These considerations have informed the development and conduct of the CLARITY trial, including the comparative effectiveness nature of the trial, the low demands made on clinical staff and the minimisation of trial-specific in-person encounters. The broad adoption of a more deliberate approach to appropriate trial features for specific settings may foster better integration of research and clinical practice, resulting in increased evidence generation, trial efficiencies and the clinical benefits resulting from research.

## Conclusion

Despite remarkable advances in the evidence-based treatment of COVID-19, it remains unclear whether treatment with ARBs—a readily available and low-cost class of medications—can reduce COVID-19 severity in patients who are otherwise not indicated. CLARITY is a pragmatic, multi-centre randomised controlled trial designed to assess the effectiveness of ARBs in reducing disease severity in COVID-19. As one of only two large-scale ARB trials currently recruiting in the Asia-Pacific region, CLARITY will make important contributions to the global evidence base for whether this class of medications are effective in the treatment of COVID-19. If found to be effective, the implementation of ARB treatment for COVID-19 into clinical practice is likely to be rapid.

## Supplementary Information

**Additional file 1.** CLARITY Trial Team.

**Additional file 2.** CLARITY Participant Information and Informed Consent Document (in English).

**Additional file 3.** CLARITY SPIRIT Fillable Checklist.

**Additional file 4.** CLARITY Trial Results Template.

**Additional file 5.** CLARITY WHO Trial Registration Data.

## Data Availability

A pre-formatted template has received pre-approval by the relevant Ethics committees to allow rapid dissemination of results to surviving participants at the conclusion of the trial (Supplementary Material—CLARITY Trial Results Template). The results of the trial will be submitted for publication in international peer-reviewed journals and for presentation at national and international conferences. Surviving participants will be individually notified of trial findings. The final data set will be under the custodianship of the Chair of the Trial Steering Committee. All individual participant data that are collected during the trial will be de-identified. The researchers intend deidentified data will contribute to global learnings through an appropriately constituted individual participant data level collaboration. Requests for data access or analysis proposals will be reviewed by the Trial Steering Committee, who will assess proposals according to criteria based on scientific merit and contribution to global knowledge. Data sharing will be performed in compliance with local Data Protection Laws. The study protocol, statistical analysis plan, and informed consent form will be publicly available prior to the completion of the trial.
