# Supplementary material for: Protocol for the Controlled evaLuation of Angiotensin Receptor blockers for COVID-19 respIraTorY disease (CLARITY): a randomised controlled trial
Source: Trials. 2021 Aug 28;22:573. doi: 10.1186/s13063-021-05521-0 (PMC8397850; doi:10.1186/s13063-021-05521-0)
Supplement: Supplementary file 1 — Additional file 1. CLARITY Trial Team. [file 13063_2021_5521_MOESM1_ESM.pdf]

CLARITY Trial Team

Steering Committee Executive: Meg Jardine (Chair), Vivekanand Jha (Co-Chair), Abhinav Bassi, Louise Burrell, Carinna Hockham, Christine Jenkins, Sradha Kotwal, Carol Pollock, Angus Ritchie, Arlen Wilcox

Steering Committee Members: Ashfak Bangi, Ashish Bhalla, Jenny Heng-Chen Chen, Sanjay D'Cruz, Michael Dymock, Simon Finfer, Greg Fox, Mayur Garg, Harry Gibbs, Lalit Gupta, Santosh Kumar Nag, Mark Jones, Benjamin Kwan, Angela Makris, George Mangos, Jennifer Martin, James McGree, Andrew McLachlan, Matthew O'Sullivan, Eugenia Pedagogos, Jeffrey Post, Vinay Rathore, Thomas Snelling, Louisa Sukkar, Richard Sullivan, Gian Luca Di Tanna, Jason Trubiano, Sophia Zoungas

Study Clinician: Sradha Kotwal

Scientific Leads: Abhinav Bassi, Carinna Hockham, Sradha Kotwal

Project Manager: Arlen Wilcox

Central Management Team: Grace Balicki, Nikita Bathla, Alison Coenen, Sedricx Fontanilla, Enmoore Lin, Martyn Ralph, Nuria Zamora

Statistical Team: James McGree, Mark Jones, Tom Snelling

Independent Data Monitoring Committee: Katherine Tuttle (Chair), Jonathan Craig, Stephane Heritier, Allison Lambert

Consumer and Community Engagement Committee: David Morgan and others

Collaborating Sites and Investigators:

Site	Location	Principle Investigator	Co-Investigator (Co-I) / Study Coordinator (SC)
**All India Institute of Medical Science, Raipur**	Chhattisgarh, India	Vinay Rathore	Rajendra Kumar Sahu (SC)
**Christian Hospital, Nabarangpur**	Odisha, India	Santosh Kumar Nag	Michael John (Co-I)
**Government Medical College and Hospital**	Chandigarh, India	Sanjay D'Cruz	Yuvraj Singh Cheema (Co-I)
**Kasturba Medical College**	Karnataka, India	Indu Rao	Afsal PM (Co-I)
**Maulana Azad Medical College and Lok Nayak Hospital**	New Delhi, India	Lalit Gupta	Lovenish Bains (Co-I)
**Postgraduate Institute of Medical Education & Research**	Chandigarh, India	Ashish Bhalla	Deepak Sharma (SC)
**Jeevan Rekha Multispecialty Hospital**	Maharashtra, India	Ashfak Bangi	
**Royal Prince Alfred Hospital**	New South Wales (NSW), Australia	Greg Fox	Nuria Zamora (SC)
**Prince of Wales Hospital**	NSW, Australia	Jeffrey Post	Kristen Overton (SC)
**Royal North Shore Hospital**	NSW, Australia	Carol Pollock	Dawn Ngai (SC)<br>Helen Clayton (SC)<br>Martyn Ralph (SC)
**St George Hospital**	NSW, Australia	Richard Sullivan	Sharon Robinson (SC)
**Concord Repatriation General Hospital**	NSW, Australia	Angus Ritchie	Samantha Hand (SC)<br>Yennie Huynh (SC)<br>Nuria Zamora (SC)
**Canterbury Hospital**	NSW, Australia	Louisa Sukkar	Nuria Zamora (SC)
**Wollongong Hospital**	NSW, Australia	Jenny Chen	Yulan Shen (SC)
**Sutherland Hospital**	NSW, Australia	Benjamin Kwan	William Lin (SC)
**Westmead Hospital**	NSW, Australia	Matthew O'Sullivan	Neela Joshi (Sc)
**Liverpool Hospital**	NSW, Australia	Angela Makris	Deepa Francis (SC)
**John Hunter Hospital**	NSW, Australia	Jennifer Martin	Martyn Ralph (SC)
**Austin Hospital**	Victoria, Australia	Jason Trubiano	Fiona James (SC)
**Sunshine Hospital**	Victoria, Australia	Eugenia Pedagogos	Shannon Kokoszka (SC)
**The Alfred Hospital**	Victoria, Australia	Harry Gibbs	Vathy Nagalingam (SC)
**Northern Health**	Victoria, Australia	Mayur Garg	Nicola Dalgleish (SC)
