# Supplementary material for: Protocol for the Controlled evaLuation of Angiotensin Receptor blockers for COVID-19 respIraTorY disease (CLARITY): a randomised controlled trial
Source: Trials. 2021 Aug 28;22:573. doi: 10.1186/s13063-021-05521-0 (PMC8397850; doi:10.1186/s13063-021-05521-0)
Supplement: Supplementary file 2 — Additional file 2. CLARITY Participant Information and Informed Consent Document (in English). [file 13063_2021_5521_MOESM2_ESM.pdf]

Figure 2: Participant Assessments

Time Point	S	B	1º Follow Up & Treatment Period															2º Follow Up
Day	<B	0	1	2	3	4	5	6	7	8	9	10	11	12	13	14	15-28	29-90
Eligibility	X																	
Consent	X																	
Demographics[1]		X																
Medical History		X																
Randomisation		X																
Concomitant Medication		X	X	X	X	X	X	X	X	X	X	X	X	X	X	X	X	
Intervention Supply		X																
Health Status Assessment[2]		X	X	X	X	X	X	X	X	X	X	X	X	X	X	X	X	X
Medication Adherence		X	X	X	X	X	X	X	X	X	X	X	X	X	X	X	X	
Blood Pressure		X	X	X	X	X	X	X	X	X	X	X	X	X	X	X		
Blood Pathology[3]		X	X		X		X		X		X					X		
Hospitalisation & Mortality																		X

S = Screening, B = Baseline, 1º = Primary, 2º = Secondary

[1] Demographics includes: Date of Birth, Sex, Ethnicity, Weight, Height, Smoking Status, COVID-19 diagnosis, comorbidities

[2] Health Status Assessment includes: Hospital Admission status, Ventilation status, Supplemental Oxygen Status, Intensive Care Unit Admission status, Mortality status, Dialysis Status & Acute Kidney Injury status

[3] Blood Pathology includes: Potassium, Serum Creatinine, creatinine kinase, estimated glomerular filtration rate, white cell count, lymphocytes, D-dimer, C-reactive protein
