# Supplementary material for: Protocol for the Controlled evaLuation of Angiotensin Receptor blockers for COVID-19 respIraTorY disease (CLARITY): a randomised controlled trial
Source: Trials. 2021 Aug 28;22:573. doi: 10.1186/s13063-021-05521-0 (PMC8397850; doi:10.1186/s13063-021-05521-0)
Supplement: Supplementary file 4 — Additional file 4. CLARITY Trial Results Template. [file 13063_2021_5521_MOESM4_ESM.pdf]

Dear CLARITY Participant,

You are receiving this letter because, between *MMM/YYYY* and *MMM/YYYY*, you took part in the Controlled evaLuation of Angiotensin Receptor Blockers for COVID-19 respIraTorY disease (CLARITY) trial, a research study designed to test whether taking angiotensin receptor blockers reduces the severity of infection, within patients diagnosed with COIV-19. It would not have been possible to find evidence for this without participants like you. **Thank you for taking part**! You have helped researchers and your doctors to learn more about how angiotensin receptor blockers can be used to improve the health of people with COVID-19.

X participants contributed to the CLARITY trial over X months. The trial is now finished, and we would like to share the results with you.

Why was the trial performed?

Before this trial, angiotensin receptor blockers were, and remain to be, a group of medications widely used to treat conditions including high blood pressure, kidney disease and heart disease. However, there was evidence to suggest that angiotensin receptor blockers may be able to limit the effect of the COVID-19 virus on the body.

What were the trial findings?

POSITIVE OUTCOME (only used if outcome is positive)

The CLARITY trial showed that **angiotensin receptor blockers reduce the severity of infection, in people diagnosed with COVID-19. Specifically, we found that people with COVID-19 who took angiotensin blockers were x% more likely to have a less severe of infection.**

These findings mean that, in the future, we can use angiotensin receptor blockers to keep people diagnosed with COVID-19 healthier.

NEUTRAL OUTCOME (only used if outcome is neutral)

Thanks to your generous participation, we found that **angiotensin receptor blockers have no effect on the severity of infection in people with COVID-19.**

These findings mean that your doctor can continue to use angiotensin receptor blockers as they normally would, knowing they will not affect outcomes in COVID-19 either way.

NEGATIVE OUTCOME (only used if outcome is negative)

Thanks to your generous participation, we found that **angiotensin receptor blockers in fact increases severity of infection in people with COVID-19.** Specifically, we found that **people with COVID-19 infection who took angiotensin receptor blockers were x% more likely to have a more severe infection.**

These findings mean that your doctor is less likely to prescribe angiotensin receptor blockers to individuals with COVID-19 infection. Whilst there may be some instances where angiotensin receptor blockers are appropriate, your doctor is now better informed to know when and when not to prescribe them.

What does this mean for me?

POSITIVE OR NEUTRAL

Every individual is different and has their own particular medical circumstances. COVID-19 can be a complex and severe infection. In any individual circumstance, many viral factors and treatments will have contributed to the outcome which is why trials are necessary to find out which treatments are effective overall. The results of CLARITY will help doctors manage people in the future with COVID-19.

Where can I learn more about this study?

The results of the trial will be published, including in scientific journals. Your doctor will have access to these. The results will also be available at [url].
