# Supplementary material for: Protocol for the Controlled evaLuation of Angiotensin Receptor blockers for COVID-19 respIraTorY disease (CLARITY): a randomised controlled trial
Source: Trials. 2021 Aug 28;22:573. doi: 10.1186/s13063-021-05521-0 (PMC8397850; doi:10.1186/s13063-021-05521-0)
Supplement: Supplementary file 5 — Additional file 5. CLARITY WHO Trial Registration Data. [file 13063_2021_5521_MOESM5_ESM.pdf]

# CLARITY WHO Trial Registration Data Set

**Primary Registry and Trial Identifying Number**

ClinicalTrials.gov

Identifier: NCT04394117

**Date of Registration in Primary Registry**

May 19, 2020

**Secondary Identifying Number**

11052020

**Source of Monetary or Material Support**

CLARITY is funded by a grant from the National Health and Medical Research Council's (NHMRC) Medical Research Future Fund (MRFF) – 2020 Respiratory Medicine Clinical Trials Research on COVID-19 Grant Opportunity.

**Primary Sponsor**

The George Institute

**Secondary Sponsor**

University of Sydney NHRMC Clinical Trials Centre

**Contact for Public Queries**

Arlen Wilcox

+61 2 9562 5203

arlen.wilcox@sydney.edu.au

**Contact for Scientific Queries**

Meg Jardine

+61 2 9562 5000

meg.jardine@sydney.edu.au

**Public Title**

CLARITY

**Scientific Title**

Controlled evaLuation of Angiotensin Receptor Blockers for COVID-19 respIraTorY Disease

**Countries of Recruitment**

India & Australia

**Health Conditions or Problems Studied**

SARS-CoV-2 COVID-19

**Interventions**

Active Comparator: Standard Care + Angiotensin Receptor Blocker (ARB)

Participants will receive an Angiotensin Receptor Blocker on top of the standard care provided by their institution.

Placebo Comparator: Standard Care + Placebo

Participants will receive a placebo on top of the standard care provided by their institution.

**Key Inclusion and Exclusion Criteria**

Inclusion Criteria:

Potential participants must satisfy all of the following:

1. Laboratory-confirmed* diagnosis of SARS-CoV-2 infection within 10 days prior to randomisation
2. Age ≥ 18 years
3. a) Systolic Blood Pressure (SBP) ≥ 120 mmHg OR b) SBP ≥ 115 mmHg and currently treated with a non-RAASi Blood Pressure (BP) lowering agent that can be ceased
4. Participant and treating clinician are willing and able to perform trial procedures.

5. Either Intended for hospital admission for management of COVID-19, or (In Australia Only) Intended for management at home with one or more of the following criteria:
    a) Age≥60 years
    b) BMI ≥30kg/m2 (derived from the patient's self-report of their height and weight where these are not measured directly)
    c) Diagnosis of diabetes defined as HbA1c ≥7% and/or the consumption of glucose lowering medication
    d) History of cardiovascular disease
    e) History of chronic respiratory illness
    f) Currently treated with immunosuppression

Exclusion Criteria:

1. Currently treated with an ACEi, ARB or aldosterone antagonist, aliskiren, or angiotensin receptor-neprilysin inhibitors (ARNi)
2. Serum potassium > 5.2 mmol/L or no potassium testing within the last 3 months
3. For those intended for hospital admission, an estimated Glomerular Filtration Rate (eGFR) <30ml/min/1.73m2 or no eGFR testing within the last 3 months, or For those intended for management at home (Australia only), an eGFR <45ml/min/1.73m2 or no eGFR testing within the last 3 months
4. Known symptomatic postural hypotension
5. Known biliary obstruction, known severe hepatic impairment (Child-Pugh-Turcotte score 10-15) - see Table below
6. Intolerance of ARB
7. Pregnancy or risk of pregnancy, defined as;
    a) (In Australia only) Women younger than 51 years who have not had a negative pregnancy test during the past 3 days and/or who do not agree to use adequate contraception
    b) (In India Only) Women who are pregnant
8. Women who are currently breastfeeding
9. Individuals who are not able to take medications by mouth at enrolment, or who are not expected to be able to take medications by mouth during the first 48 hours after randomisation

**Study Type**

Type: Interventional

Allocation: Randomized (1:1 ratio, stratified according to country and whether the participant is planned for hospital admission or home-based care)

Intervention Model: Parallel Assignment

Masking: Single (Outcomes Assessor)

Masking Description: Trial Statistician and sponsor staff will remain blinded to treatment allocation throughout the trial.

Primary Purpose: Treatment

Phase: Phase 4

**Date of First Enrollment**

June 19, 2020

**Sample Size**

Estimated Total Enrolment: 1500

Enrolment To Date: 700

**Recruitment Status**

Recruiting: participants are currently being recruited and enrolled.

**Primary Outcomes**

7-Point National Institute of Health Clinical Health Score [ Time Frame: 14 Days ]

To determine whether the addition of the intervention, compared to standard care, changes the clinical health score of a participant on the following scale;

1. Not hospitalized, no limitations on activities.
2. Not hospitalized, limitation on activities;
3. Hospitalized, not requiring supplemental oxygen;
4. Hospitalized, requiring supplemental oxygen;
5. Hospitalized, on non-invasive ventilation or high flow oxygen devices;
6. Hospitalized, on invasive mechanical ventilation or extracorporeal membrane oxygenation (ECMO);
7. Death;

**Key Secondary Outcomes**

1. 7-Point National Institute of Health Clinical Health Score [ Time Frame: 28 Days ]
   To determine whether the addition of the intervention, compared to standard care, changes the clinical health score of a participant on the following scale;

   a) Not hospitalized, no limitations on activities.
   b) Not hospitalized, limitation on activities;
   c) Hospitalized, not requiring supplemental oxygen;
   d) Hospitalized, requiring supplemental oxygen;

e) Hospitalized, on non-invasive ventilation or high flow oxygen devices;
f) Hospitalized, on invasive mechanical ventilation or extracorporeal membrane oxygenation (ECMO);
g) Death;

2. Mortality [ Time Frame: 28 Days ]
   To determine whether the addition of the intervention, compared to standard care, changes the risk of all cause mortality

3. Mortality [ Time Frame: 90 Days ]
   To determine whether the addition of the intervention, compared to standard care, changes the risk of all cause mortality

4. Intensive Care Unit Admission [ Time Frame: 28 Days ]
   To determine whether the addition of the intervention, compared to standard care, changes the count of all cause Intensive Care Unit admission

5. Intensive Care Unit Admission [ Time Frame: 90 Days ]
   To determine whether the addition of the intervention, compared to standard care, changes the count of all cause Intensive Care Unit admission

6. Intensive Care Unit Number of Days [ Time Frame: 90 Days ]
   To determine whether the addition of the intervention, compared to standard care, changes the number of days total, of intensive care unit admission

7. Respiratory Failure [ Time Frame: 28 Days ]
   To determine whether the addition of the intervention, compared to standard care, changes the incidence of respiratory failure

8. Dialysis Requirement [ Time Frame: 28 Days ]
   To determine whether the addition of the intervention, compared to standard care, changes the requirements for dialysis

9. Hospitalisation Days [ Time Frame: 28 Days ]
   To determine whether the addition of the intervention, compared to standard care, changes the number of hospitalisation days

10. Hospitalisation Days [ Time Frame: 90 Days ]
    To determine whether the addition of the intervention, compared to standard care, changes the number of hospitalisation days

11. Ventilator-Free Days [ Time Frame: 28 Days ]
    To determine whether the addition of the intervention, compared to standard care, changes need for ventilation

12. Dialysis Days [ Time Frame: 28 Days ]
    To determine whether the addition of the intervention, compared to standard care, changes need for dialysis

13. Acute Kidney Injury [ Time Frame: 28 Days ]
    To determine whether the addition of the intervention, compared to standard care, changes risk of acute kidney injury, based on the idney Disease: Improving Global Outcomes definition

14. Hypotension Requiring Vasopressors [ Time Frame: 28 Days ]
    To determine whether the addition of the intervention, compared to standard care, changes risk of hypotension requiring vasopressors

Other Outcome Measures:

1. Hyperkalaemia [ Time Frame: Day 28 ]
   To determine whether the addition of the intervention, compared to standard care, changes risk of hyperkalaemia.

2. Oxygen Saturation [ Time Frame: Day 28 ]
   To determine whether the addition of the intervention, compared to standard care, changes risk of decreased oxygen saturation

3. Oxygen Saturation [ Time Frame: Day 14 ]
   To determine whether the addition of the intervention, compared to standard care, changes risk of decreased oxygen saturation

**Ethics Review**

Approved by The George Institute for Global Health Ethics Committee in India (ref. no. 14/2020) on June 23, 2020.

Contact Details:

The George Institute Ethics Committee

+91 11 4158 8091-93

info@gerogeinstitute.org.in

Approved by Sydney Local Health District Ethics Review Committee (Royal Prince Alfred Hospital Zone) (Code: EC0113) in Australia (ref. no. X20-0118 & 2020/ETH00742) on April 28, 2020.

Contact Details:

Research Ethics and Governance Office

Royal Prince Alfred Hospital

(02) 9515 6766

SLHD-RPAEthics@health.nsw.gov.au

**Completion Date**

Estimated Study Completion Date: August 30, 2021

**Summary Results**

N/A

**IPD Sharing Statement**

For researchers outside the trial, data extracts may be made available upon request to the TSC, who will facilitate a role as a review board to assess proposals based on sound science, benefit-risk balancing and research team expertise. Appropriate data will be made available to approved proposals. This process will be in effect for a period following primary publication of the domain-specific trial results. Appropriate data shared will only be done so in a de-identified manner and will require separate ethical approval from the HREC.
